# Unusual and High Origin of Terminal Branches of the Brachial Artery: A Cadaveric Study With Embryological and Clinical Correlations

**DOI:** 10.7759/cureus.110401

**Published:** 2026-06-07

**Authors:** Vinay Sharma, Aruna Arya, Padamjeet Panchal, CS Ramesh Babu

**Affiliations:** 1 Anatomy, Muzaffarnagar Medical College, Bahadarpur, IND; 2 Anatomy, All India Institute of Medical Sciences Patna, Patna, IND

**Keywords:** anatomical variation, brachial artery, cadaveric study, high bifurcation, high-origin radial artery, superficial ulnar artery, upper limb

## Abstract

Background: The brachial artery constitutes the dominant arterial supply of the upper extremity and undergoes terminal division into the radial and ulnar arteries within the cubital fossa at the level of the radial neck. Deviations from this classic bifurcation pattern, whether in its location or configuration, carry substantial implications for both anatomical knowledge and clinical practice.

Objectives: To systematically document and characterize instances of anomalous, proximally displaced terminal branches of the brachial artery observed during routine anatomical dissection and to interpret these findings within their embryological context alongside their implications for contemporary clinical and surgical practice.

Materials and methods: A retrospective observational investigation was conducted on 116 upper extremities from 58 embalmed adult cadavers over a continuous 14-year period (2011-2025), conducted jointly at two academic anatomy departments. All arterial structures, from the axilla through to the cubital fossa and distal forearm, were methodically exposed and catalogued.

Results: A total of 9 (7.7%) limbs demonstrated anomalous high-origin superficial terminal branches. This comprised two instances of a bilateral superficial ulnar artery, each arising from the axillary artery rather than the expected brachial trunk, and seven instances of a proximally originating superficial radial artery (right side [five] and left side [two]). In every case identified, the variant vessel coursed anterior to the pronator teres muscle. All seven radial artery cases demonstrated a morphologically consistent finding of proximal tortuosity and segmental dilatation, likely related to hemodynamic alterations induced by passage through a fibro-osseous tunnel formed by the bicipital aponeurosis.

Conclusions: This investigation describes a bilaterally symmetrical axillary origin of the superficial ulnar artery, an exceptionally rare anatomical configuration, along with a proximal radial artery origin concordant with published global estimates. Recognition of these variants is indispensable for the safe conduct of venepuncture, arterial cannulation, cardiac catheterization via the transradial route, and reconstructive or orthopedic procedures involving the upper extremity.

## Introduction

The brachial artery (BA) navigates the arm as the direct continuation of the axillary artery below the inferior border of the teres major and terminates at the radial neck level in the cubital fossa by bifurcating into the ulnar and radial arteries [1]. The BA terminal division, topographically corresponding to or immediately distal to the antecubital crease, is regarded as one of the more reliable vascular reference points in upper limb anatomy. Nevertheless, substantial deviation from this expected arrangement has been well-recognized, encompassing variation in both the level and configuration of the bifurcation, with important ramifications for diagnostic and operative practice.

Anatomists and clinicians have described the branching behavior of BA for several centuries. Case-level documentation of such anomalies can be traced back to the seventeenth century, though systematic classification did not emerge until Quain's seminal contribution in 1844 [2]. The brachial artery is the most frequently implicated vessel in upper limb arterial anomalies, with at least six distinct branching configurations recognized. In a systematic review and meta-analysis of 57 studies encompassing 12,090 upper limbs. Across 48 studies (10,643 limbs), BA variations were identified in 1,064 limbs (10%), suggesting that brachial artery variation is a universal anatomical trait rather than a population-specific one. The brachioradial artery, the most frequent BA variant, was present in 598 (6%) of 9,971 limbs across 40 studies. The superficial brachial artery (SBA) was reported across 17 studies (4,258 limbs), with a pooled prevalence of 256 (6%) limbs [1]. Tsakotos et al. reported a rare cadaveric case of high axillary artery bifurcation at the lower border of the 2nd rib into a superficial axillary stem and a deep axillary stem. The superficial axillary stem continued as a superficial brachial artery [3]. Among the most commonly encountered variants is a proximally situated bifurcation, sometimes extending as far proximally as the axilla, which divides into radial and ulnar trunks, an anomalous superficial BA, or an early departure of the radial artery known as the brachioradial artery (BRA), with the ulnar and common interosseous arteries persisting as a shared trunk [3-6]. The frequency of high division of the brachial artery warrants heightened awareness in both operative and diagnostic settings, as unrecognized variants may predispose to iatrogenic injury, catheterization failure, and misinterpretation during angiographic procedures [3,7].

The embryological underpinnings of these variations have been well characterized. Upper limb arterial morphogenesis is initiated by the axis artery, which originates from the lateral branch of the seventh intersegmental artery derived from the dorsal aorta [8]. The central axis artery differentiates to form the axillary artery, the BA, the anterior interosseous artery, and the deep palmar arch [4]. A superficial BA also participates as a key transient vessel during normal upper limb vascular development; arterial variants arise when portions of the embryonic capillary plexus persist, expand, or undergo unexpected differentiation rather than completing their anticipated regression [9,10]. Disturbances in this vascular morphogenetic sequence result in a spectrum of anomalies affecting both the origin and trajectory of upper-limb vessels [11,12].

The practical relevance of high-origin or superficially coursing terminal branches of the BA extends across multiple clinical disciplines and has attracted increasing scrutiny in recent years. Transradial arterial access has become the preferred route for coronary angiography and percutaneous coronary intervention worldwide; however, procedural failure related to difficulty with transradial navigation was significantly higher in those with upper-limb arterial anomalies [13,14]. Proximally situated and superficially coursing terminal branches alter the expected topographic relationship between vessels, nerves, and muscles, creating additional operative hazard during orthopedic, plastic, vascular, and reconstructive procedures at the arm and elbow [15,16]. A thorough understanding of upper limb arterial anatomy is therefore critical to minimize the risk of injury, particularly during arterial cannulation, venepuncture, arteriovenous fistula construction, and elective upper limb surgery [17,18]. Because superficially coursing arteries may be indistinguishable from veins on clinical inspection, unrecognized intra-arterial injection at the cubital fossa or forearm carries the potential for devastating consequences, including thrombosis, tissue gangrene, and, in extreme scenarios, limb amputation [2,19]. 

Despite growing clinical awareness, many such variants remain undetected until encountered unexpectedly during surgery or identified incidentally through imaging or dissection studies. Numerous investigators have advocated preoperative vascular mapping using Doppler ultrasonography or angiography as standard practice whenever a variant is suspected, particularly in the context of vascular access procedures, arteriovenous fistula creation, or surgery of the elbow and forearm [1,20,21]. The present report aims to characterize unusual and proximally displaced terminal branches of the BA identified during routine cadaveric dissection over 14 years, to elucidate their embryological basis, and to contextualize their clinical significance within modern surgical and interventional practice.

## Materials and methods

This investigation was a retrospective study. It was conducted as a collaborative effort between two anatomical departments: the Department of Anatomy, Goldfield Institute of Medical Sciences and Research, and the Department of Anatomy, Muzaffarnagar Medical College, Muzaffarnagar. Data were collected from the anatomy department's repository of preserved specimens.

The study material comprised 116 upper extremities obtained from 58 formalin-embalmed adult human cadavers of both sexes, sourced through the respective institutional body donation programs in accordance with the provisions of the Anatomy Act. All specimens had been preserved in formalin and used for routine undergraduate teaching dissection.

Upper limb and axillary dissections were performed systematically in accordance with established anatomical dissection protocols. The BA and its terminal branches were carefully exposed and followed along their complete course from the axillary region to the cubital fossa and into the forearm. Special attention was paid to detecting any anomalous site of origin, atypical course, or aberrant terminal branching configuration. For each limb, the precise level at which the BA was divided into radial and ulnar arteries was recorded. Concomitant vascular anomalies, including accessory vessels and atypical relationships between arteries and adjacent neural or muscular structures, were also documented where encountered.

All anomalous findings were photographed in situ before proceeding with further dissection to ensure comprehensive and accurate documentation. The collated data were systematically compiled, tabulated, and analyzed to derive the study's conclusions. Findings were interpreted in light of the contemporary literature on upper limb arterial variation, with emphasis on their embryological basis and clinical relevance.

## Results

In total, nine instances of anomalous high-origin superficial terminal branches of the BA were identified across the 116 limbs dissected, corresponding to an overall prevalence of 7.7%. These comprised two limbs (1.72%) with a high origin of the superficial ulnar artery (HOSUA) and seven limbs (6.03%) with a high origin of the superficial radial artery (HOSRA).

High origin superficial ulnar artery (HOSUA)

**Figure 1:**
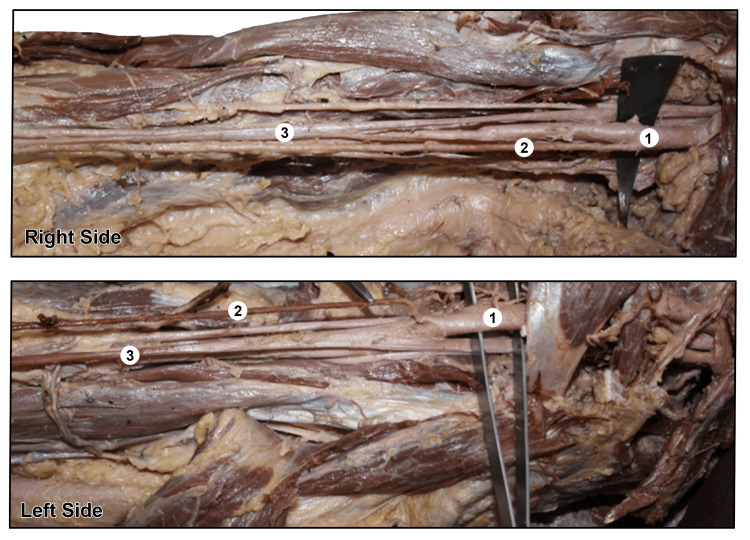
A high origin of the superficial ulnar artery (SUA) was identified bilaterally in one cadaver. 1. Axillary artery, 2. High-origin superficial ulnar artery (HOSUA), 3. formation of the median nerve.

Both HOSUA cases were identified in the right and left upper limbs of a single cadaveric specimen, representing a bilateral presentation. In each instance of HOSUA, the SUA originated from the anterior surface of the axillary artery at a level proximal to the convergence of the median nerve roots and then coursed superficially through the arm and forearm, anterior to the muscular plane. The combination of bilateral presentation and axillary origin renders this an exceedingly rare anatomical configuration (Figure 1).

Left Upper Limb

The HOSUA originated from the anterior surface of the axillary artery at a point between the lateral and medial contributions to the median nerve before they coalesced into a single trunk. Following its origin, the vessel ran anteriorly and superficially over the biceps brachii muscle, maintaining this superficial course through the cubital fossa. It then continued anterior to the superficial head of the pronator teres muscle, traversing the forearm superficial to the muscular plane throughout. Distally, the vessel descended deep and medial to the palmaris longus tendon to participate in the formation of the superficial palmar arch. Meanwhile, the residual BA was observed to terminate by dividing into the radial artery and a common interosseous artery. Accordingly, three arterial channels were distinguishable within the forearm: the SUA, the radial artery, and the common interosseous/median artery, with the latter pair representing the terminal branches of the residual brachial trunk.

Right Upper Limb

The HOSUA on the right side arose from the anterior aspect of the axillary artery at a comparable level, again before the formation of the median nerve. Its subsequent course along the medial arm was markedly superficial, lying anterior to the main neurovascular bundle. A particularly notable observation was that this artery occupied the same tissue plane as the superficial venous network. The downstream course of the artery through the cubital fossa and forearm paralleled that described on the left side.

The bilateral occurrence of HOSUA originating from the axillary artery in a single cadaver constitutes an exceptionally uncommon finding. While HOSRA variants are comparatively well represented in the anatomical literature, HOSUA remains a considerably rarer entity; its bilateral presentation from an axillary source in a single specimen is of particular anatomical and clinical importance.

High-origin superficial radial artery (HOSRA)

Seven cases of HOSRA were identified, with a right-sided predominance (five right limbs, two left limbs). No single cadaver demonstrated a bilateral HOSRA.

**Figure 2:**
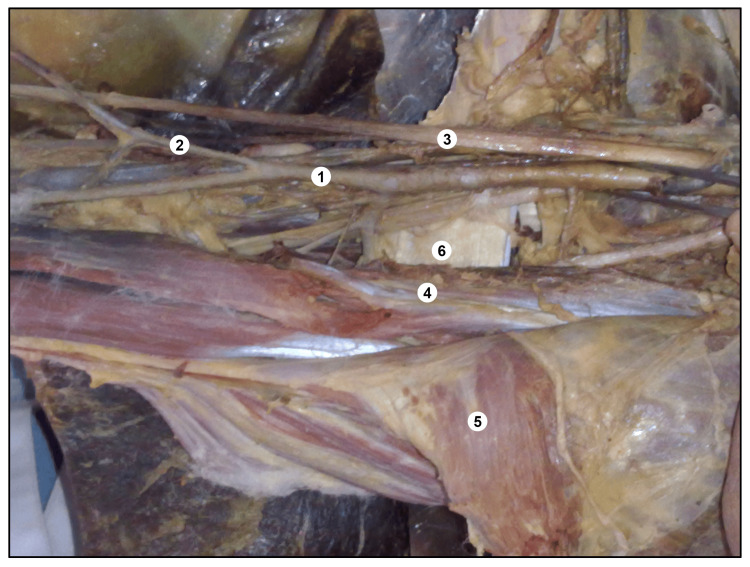
A cadaver showing the left-side high origin of the superficial radial artery. 1. Brachial Artery, 2. High-origin superficial radial artery, 3. Median nerve, 4. Bicep brachii, 5. Pectoralis major, 6. Teres major.

The level at which the high radial artery departed from the parent arterial trunk exhibited marked variability across cases. The origin ranged from as far proximally as the commencement of the brachial artery at the lower margin of the teres major to as distally as just proximal to the cubital fossa (Figure 2).

**Figure 3:**
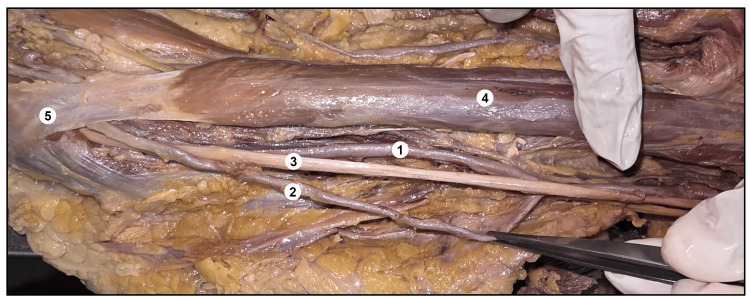
A cadaver showing the right-sided high origin of the superficial radial artery. 1. Brachial artery, 2. High origin of the superficial radial artery, 3. Median nerve, 4. Biceps brachii, 5. Bicipital aponeurosis.

Across all seven cases, a single morphological feature was uniformly identified, whereby the high-origin radial artery was consistently observed to pass beneath the bicipital aponeurosis. More precisely, a fibro-muscular tunnel-like passage formed between the bicipital aponeurosis and the tendon of the biceps brachii was consistently traversed by the high-origin radial artery. In all cases, the segment of the artery immediately proximal to this tunneled region displayed tortuosity and luminal dilatation (Figure 3).

**Figure 4:**
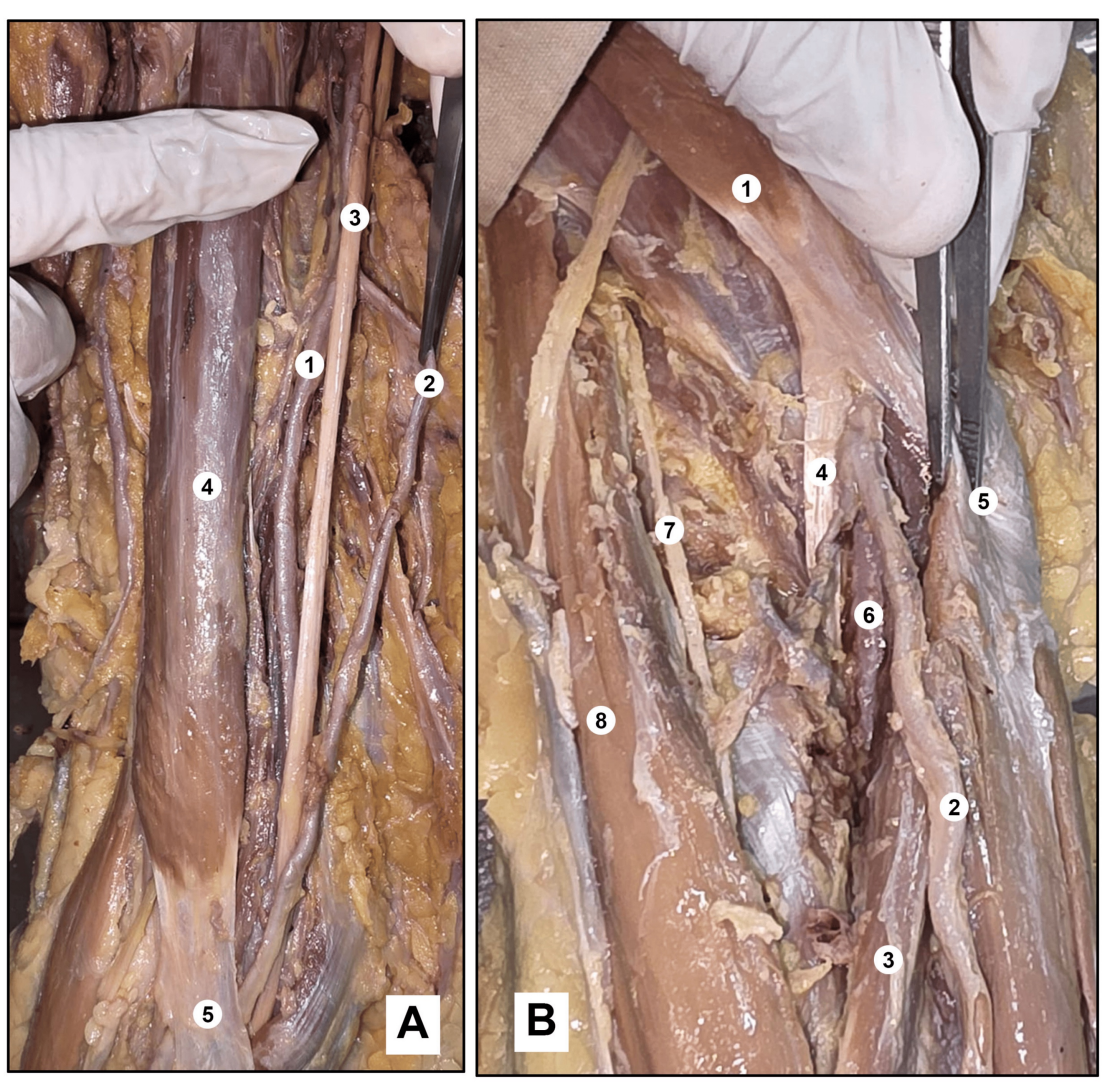
Showing (A) Tortuous course of the superficial radial artery in the lower part of the arm, proximal to the cubital fossa. (B) The superficial radial artery coursing superficial to the pronator teres and entering the forearm. (A) 1. Brachial artery, 2. Superficial radial artery, 3. Median nerve, 4. Biceps brachii, 5. Bicipital aponeurosis. (B) 1. Biceps brachii, 2. Superficial radial artery, 3. Pronator teres, 4. Tendon of biceps brachii, 5. Bicipital aponeurosis, 6. Median artery, 7. Radial nerve, 8. Brachioradialis muscle.

These morphological features may be considered in relation to the hemodynamic effects induced by extrinsic compression at this anatomical point of constriction. The tortuosity of the high-origin radial artery may have resulted from altered flow dynamics in the immediately proximal arterial segment due to compression (Figure 4).

In limbs where the radial artery arose at the mid-arm level or at the very origin of the BA, a characteristic spiraling relationship with the median nerve was documented. In these cases, the artery initially occupied a deep and lateral position relative to the median nerve in the proximal arm; it subsequently crossed around the nerve to assume a medial and superficial position in the mid-arm before reverting to a lateral relationship with the nerve as it approached the cubital fossa, thus completing a spiral course around the median nerve during its descent.

**Figure 5:**
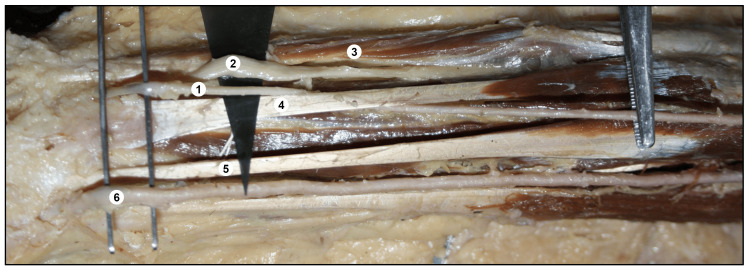
The superficial ulnar artery runs lateral and superficial to the deep and medial to the tendon of the palmaris longus. 1. Superficial ulnar artery, 2. Ulnar nerve, 3. Flexor carpi ulnaris, 4. tendon of palmaris longus, 5. Flexor carpi radialis, 6. Radial artery.

A consistent and unifying observation across all nine anomalous cases, irrespective of whether the proximally displaced vessel was ulnar or radial in type, was that the variant artery invariably coursed anterior to the pronator teres muscle. Simultaneously, the residual BA in each case proceeded to its usual division into the common interosseous artery and its companion vessel, thereby sustaining deep forearm perfusion. The anomalous superficial vessel and the residual brachial terminal branches each converged toward their expected wrist-level positions, reconstituting a functionally normal distal vascular arrangement (Figure 5).

## Discussion

Variant anatomy of BA is among the more clinically consequential findings encountered by anatomists, radiologists, and operating surgeons alike [1]. The brachial artery is the most frequently implicated vessel in upper limb arterial anomalies, with deviation rates of approximately 1,064 (10%) limbs reported across 48 studies, with a proximally displaced terminal bifurcation constituting one of the predominant patterns of variation [1]. The overall prevalence of 9 (7.7%) documented in the present study falls within the lower bound of estimates reported in international literature. A published cadaveric series of 34 limbs by Buckway et al. has reported an isolated high bifurcation in up to 4 limbs (11.7%) and a combination of high bifurcation with a concurrent SUA in 1 (2.94%) limb [19]. The prevalence identified in the present study falls within the lower range of published estimates. This may reflect genuine population-level differences in the frequency of such variants. It is also worth noting that angiographic investigations have consistently reported slightly lower rates than cadaveric dissection studies. This discrepancy is plausibly attributable to selection bias, as patients undergoing angiography represent a clinically selected subgroup and therefore may not accurately reflect the true prevalence of such variants in the general population [22].

High-origin superficial ulnar artery (HOSUA)

The SUA was identified as an uncommon variant, occurring in 16 (4.2%) of 384 upper limbs. Of these, four (25%) originated from the axillary artery and 12 (75%) from the brachial artery (BA). Among the 12 cases arising from the BA, the SUA originated from the upper one-third of the BA in six cases (50%), from the inferior one-third of the BA in three cases (25%), and from the superficial brachial artery in the remaining three cases (25%) [10]. The HOSUA originates in the right arm from the second part of the axillary artery and proceeds superficially in the forearm. The axillary artery coursed normally in the arm, then coursed as the brachial artery, terminating in the radial and common interosseous arteries, with the absence of a normal ulnar artery [23]. In a combined ultrasound and cadaveric dissection study of 150 upper limbs, 1 (0.66%) case was identified in which a HOSUA of axillary origin coexisted with the SRA, illustrating the particular rarity of this combined pattern [21]. The bilateral case identified in the current study is therefore exceptionally rare. A comparable bilateral presentation was reported by Troupis et al. following dissection of an elderly male cadaver, in which SUAs were bilaterally present, with axillary origins, but followed differing courses in the respective arms; a persistent median artery was also noted [24]. Sieger et al. reported that the BA bifurcated at the proximal third of the arm into a larger BRA and a smaller BUA, lateral to the median nerve. The BRA coursed laterally to the median nerve, unusually giving origin to the common interosseous artery. The BUA ran medial to the median nerve, superficial to all forearm muscles except palmaris longus, terminating as the superficial palmar arch [25]. In a separate series, Fadel and Amonoo-Kuofi documented one case of bilateral SUA's arising from the second portion of the axillary artery bilaterally, pursuing a superficial course throughout the arm and forearm and contributing dominantly to the superficial palmar arch [26]. Gupta et al. similarly reported bilateral SUA with high-level axillary origin, describing it as a clinically significant finding of major relevance to both surgical and radiological practice [27].

The specific site of origin documented in the present study, arising from the anterior axillary artery wall between the lateral and medial roots of the median nerve, before their union, carries distinct embryological and topographic significance. A comparable unilateral case was reported by Natsis et al., in which the SUA arose from the right axillary artery at the level of the junction of the two median nerve roots, pursuing a looping course that crossed the lateral root of the median nerve in the proximal two-thirds of the arm before crossing to a medial position distally; the axillary artery after this departure continued as the BA and bifurcated into the radial artery and common interosseous artery in the cubital fossa, with the normal ulnar artery absent [28]. The three-trunk arrangement observed in the present study, comprising a SUA, a radial artery, and a common interosseous/median artery representing the residual brachial terminal division, is in line with findings from multiple prior investigators [24-26].

The observation that the right SUA lies in the same anatomical plane as the superficial venous drainage is of direct clinical relevance. Distinguishing a superficial artery from an adjacent vein on physical examination alone is not consistently reliable; the absence of a palpable pulse may not be demonstrable when a tourniquet is in place [29]. In clinical practice, such a vessel is vulnerable to misidentification as a vein, with potentially serious consequences, including inadvertent intra-arterial injection and interference with the passage of an angiographic catheter [30]. Superficially located ulnar or brachio-ulnar arteries (BUA) carry a specific risk of injury; even minor local trauma may provoke significant hemorrhage, and their unrecognized cannulation during venous access attempts may precipitate tissue ischemia, necrotic change, or gangrene [19,31]. Palpation for a superficial pulse before cannulation may help prevent inadvertent arterial injury in cases of HOSUA. The clinical sequelae of intra-arterial drug or fluid injection in this setting can be severe, with documented outcomes including pseudoaneurysm formation requiring surgical resection and vascular reconstruction [31]. High bifurcation of the BA can present in various forms and has important clinical implications, including increased failure rates and reduced patency of arteriovenous fistulas used for hemodialysis [32]. Additional case reports have described inadvertent arterial cannulation of the SUA, detected only by retrograde pulsatile blood backflow into intravenous tubing, emphasizing the necessity for vigilance at any cannulation site in the cubital fossa or forearm [29].

High-origin superficial radial artery (HOSRA)

Seven cases of HOSRA were identified in the present series, with a predominance in right upper limbs (five right, two left), a laterality pattern consistent with findings from multiple prior cadaveric investigations [22]. The prevalence of a high-origin radial artery varies considerably across studies. A cadaveric study conducted in a Black Kenyan population examined 62 limbs, of which eight (12.9%) were found to have high-origin radial arteries. Among these, the majority, six (9.7%), originated as branches of the brachial artery, whereas the remaining two (3.2%) arose from the axillary artery [22]. A study on 600 Singaporean Chinese cadavers (1,200 upper limbs) identified only two (0.33%) cases of high-origin radial artery [33]. In a cadaveric study conducted by Niedenfur et al., a high-origin radial artery was identified in 39 out of 192 cadavers (20.3%), or equivalently, in 53 out of 384 upper limbs (13.8%) [9]. A cadaveric study conducted by Naseem et al. on 31 donor bodies evaluated 61 upper limbs for BA variations. A high-origin radial artery was identified in seven (11.5%) of 61 upper limbs. All seven cases originated from the axillary artery, with four (57.1%) on the right and three (42.9%) on the left [34]. The prevalence of a high-origin radial artery in the present study, seven (6.03%), broadly aligns with these published figures.

The pronounced variability in the proximal origin of the high radial artery, extending from immediately below the teres major to just proximal to the cubital fossa, has been extensively documented in the literature. Halałaj et al., in a systematic analysis of 120 upper limbs, documented the brachioradial artery arising at a mean of 178 mm proximal to the humeral intercondylar line (range 126-260 mm), with all cases demonstrating a deep passage beneath the bicipital aponeurosis within the cubital fossa [5]. Nasr provided further documentation of the range of origin and course variability of the radial artery, together with its clinical implications [6]. The uniformly present finding in the current series of proximal radial artery tortuosity and dilatation at the bicipital aponeurosis is consistent with the observation in the literature that a high radial artery origin predisposes to the development of tortuosity [5]. At the same time, dedicated cadaveric case reports have described a kinked and tortuous course in mid-arm, high-origin radial arteries that may predispose to thrombosis, vasospasm, or occlusion, with downstream forearm and hand ischemia [35]. The compressive hemodynamic effect exerted by the bicipital aponeurosis on the high origin of the radial artery constitutes a plausible explanation for the consistently observed pattern of proximal dilatation and vessel tortuosity in the present series.

The spiral relationship between the high-origin radial artery and the median nerve is a well-recognized morphological feature. It commences in a deep lateral position, transitioning to a medial superficial position in the mid-arm, and resumes a lateral orientation at the cubital fossa. In previously reported cases of the brachioradial artery, the vessel has been described as running anterior to the median nerve, crossing its surface above the intercondylar line, before adopting a lateral relationship at the cubital fossa [5,36]. The more elaborate spiraling configuration observed in the present study for cases of very proximal origin reflects the anomalous vessel's longer intrabrachial course, necessitating a wider looping trajectory before aligning with its standard forearm position.

Consistent features across both variants

Irrespective of whether the anomalous vessel was an SUA or SRA and regardless of its precise level or side of origin, a shared characteristic present in all nine anomalous cases was the passage of the variant artery anterior to the pronator teres muscle. The superficial BUA and BRA courses are consistently anterior to the forearm flexor musculature [25]. The superficial BUA travels superficially relative to all forearm muscles except the palmaris longus before contributing to the superficial palmar arch; this configuration is specifically clinically relevant because the vessel may be confused with a vein, creating the risk of inadvertent arterial cannulation and subsequent limb ischemia [25,37]. The observation that in each case the residual brachial artery continued to generate a common interosseous artery, thereby sustaining deep forearm tissue perfusion, and that the anomalous vessel ultimately reached its expected wrist-level position demonstrates that the distal vascular architecture of the hand remained functionally intact, in agreement with prior reports [25,26,28].

Embryological basis

The developmental basis of the variants documented in the present study is interpretable within the established framework of upper-limb arterial morphogenesis. The brachial artery is developed from the principal arterial trunk, which arises from the lateral branch of the seventh intersegmental artery, itself derived from the dorsal aorta. It grows in proportion to the developing limb [8]. Arterial anomalies arise when segments of the primary capillary plexus persist, enlarge, and differentiate rather than undergoing the expected regression [10]. In the context of the HOSUA, failure of regression of the superficial BA results in its continuation and subsequent differentiation into the ulnar artery, yielding the pattern of proximal brachial bifurcation [9]. Persistence of the superficial BA or failure of normal anastomotic communication between the superficial brachial and the deep axial vessels may also generate HOSUA or HOSRA, at times with associated tortuosity and atypical branch configurations, including a common interosseous artery arising from the radial [6,12]. The bilateral symmetry of the anomaly observed in the present cadaver may reflect a coordinated developmental perturbation occurring during the critical embryonic window of upper limb vascular patterning; this interpretation is supported by one large series in which all female cadavers found to bear superficial BUA demonstrated the variant bilaterally, raising the possibility of a constitutional or hereditary predisposition [38].

Clinical implications

The findings of this study have wide-ranging clinical significance across multiple specialties. Misidentification of anomalous superficial vessels as veins can precipitate inadvertent intravascular injection, with the potential for tissue ischemia, necrosis, and limb loss; their unexpected presence may also complicate planned surgical procedures, including orthopedic reconstruction, vascular bypass grafting, or flap harvesting [21,39]. Proximal or mid-arm bifurcations can position arteries in atypical relationships to the median, radial, and ulnar nerves, increasing the risk of complications during arm and elbow surgery, including inadvertent laceration or hematoma formation when anomalous branches lie close to the operative field [15,16,40]. From the standpoint of interventional cardiology and vascular access, proximally displaced and superficially positioned terminal branches of the BA are associated with measurably higher failure rates in transradial coronary and neurovascular interventions. In this prospective study of 1,195 transradial procedures, upper limb arterial anomalies were identified in 117 (9.8%) patients. The HOSRA was the dominant anomaly, present in 97 (82.9%) patients. Despite this numerical dominance, HOSRA posed the least procedural challenge, and most cases were detected only on routine post-procedural angiography, meaning operators navigated through them unknowingly. Its transradial failure rate was just 4.1%. [14]. With respect to arteriovenous fistula creation for hemodialysis, a high BA bifurcation has been associated with compromised fistula patency and elevated failure rates, and current consensus recommends preoperative duplex ultrasonographic mapping as a prerequisite to identify optimal inflow vessels and avoid unnecessary reliance on secondary access configurations [41,42]. Superficially coursing variant arteries may also carry a smaller caliber than the normal main trunk, which can compromise intended cannulation and flow adequacy if inadvertently selected as the inflow vessel [43]. Intraoperative Doppler ultrasound provides a valuable adjunct for reconstructive and orthopedic surgeons, and preoperative angiographic assessment in flap surgery may allow precise delineation of the course and origin of a variant artery [44]. Multiple independent investigators have concluded that preoperative Doppler ultrasound or high-frequency ultrasound mapping is the most reliable noninvasive method for detecting upper-limb arterial variants before proceeding with vascular access procedures, arteriovenous fistula surgery, or complex upper-limb operative interventions [20,21]. Doppler ultrasonography should be adopted as a standard preoperative screening modality for patients scheduled for upper limb vascular access procedures or surgery [17,22,23].

Limitations of the study

The study's limitations include a modest sample size, which limits definitive conclusions about prevalence, particularly for the rare bilateral superficial ulnar artery finding, which was observed in only a single cadaver. The absence of demographic stratification precludes sex-based or laterality analyses, and a focus on a single northern Indian population limits generalizability. Reliance on macroscopic dissection without correlation with Doppler ultrasound or computed tomographic angiography and the lack of morphometric measurements relative to bony landmarks further constrain precise characterization and comparability with published series. Future studies combining larger multiethnic cadaveric samples with integrated radiological mapping would substantially address these shortfalls.

## Conclusions

Among 116 upper extremities examined, nine instances (7.7%) of anomalously proximal terminal branches of the BA were identified, comprising a rare bilateral HOSUA of axillary origin and seven cases of HOSRA with consistent proximal tortuosity related to passage through the bicipital aponeurosis. In all cases, distal vascular architecture remained functionally intact. These findings emphasize the clinical importance of recognizing such variants, particularly in the context of venepuncture, transradial catheterization, and arteriovenous fistula creation. Routine preoperative Doppler ultrasonographic mapping is recommended to reduce the risk of inadvertent vascular injury.
